# Mental and psychosocial health and health related quality of life before and after cardiac rehabilitation: a prospective cohort study with comparison to specific population norms

**DOI:** 10.1186/s12955-022-01994-y

**Published:** 2022-06-07

**Authors:** Felix Angst, Raoul D. Giger, Susanne Lehmann, Peter S. Sandor, Peter Teuchmann, Adam Csordas

**Affiliations:** 1 Research Department, Rehaklinik Bad Zurzach, Zurzach Care Group, Quellenstrasse 34, 5330 Bad Zurzach, Switzerland; 2 Department of Cardiology, Rehaklinik Bad Zurzach, Zurzach Care Group, Bad Zurzach, Switzerland

**Keywords:** Acute coronary syndrome, Rehabilitation, Outcome, Psycho-social, Norm, SF-36

## Abstract

**Background:**

Data on mental health improvement after cardiac rehabilitation (CR) are contradictory. The aim was to examine the mental and psycho-social health of patients admitted to our rehabilitation center following hospital treatment for acute coronary syndrome, before and after multidisciplinary CR.

**Methods:**

Outcome was measured at admission and discharge by the 36-Item Short Form Survey (SF-36), the Symptom Checklist-90 Revised (SCL-90R), the Coping Strategy Questionnaire (CSQ) and the 6-min-walking distance test. The patients’ health status was compared with norms of sex-, age- and comorbidity-matched data from the German general population. Score differences from norms were measured by standardized mean differences (SMDs); health changes were quantified by standardized effect sizes (ESs). Their importance for comprehensive assessment was quantified by explorative factor analysis.

**Results:**

Of n = 70 patients followed-up (male: 79%; mean age: 66.6 years), 79% had ≥ 3 comorbidities. At baseline, SF-36 Physical functioning (SMD = − 0.75), Role physical (− 0.90), Social functioning (SMD = − 0.44), and Role emotional (SMD = − 0.45) were significantly worse than the norm. After CR, almost all scores significantly improved by ES = 0.23 (SCL-90R Interpersonal sensitivity) to 1.04 (SF-36 Physical functioning). The strongest factor (up to 41.1% explained variance) for health state and change was the mental health domain, followed by function & pain (up to 26.3%).

**Conclusions:**

Normative deficits in physical and psycho-social health were reported at baseline. After CR, at follow-up, all scores, except phobia, showed significant improvement. The comprehensive measurement of bio-psycho-social health should not be limited to depression and anxiety but include, especially, the somatization and social participation dimensions.

## Plain language summary

Cardiac rehabilitation (CR) after a heart attack tends to focus more on patients’ physical health than on their psycho-social wellbeing. This study measured patients’ psycho-social health before and after CR, using specific self-rating instruments and the 6-min-walking-distance test and compared the results with normative data obtained from the general population. Before CR, patients’ physical and social function and role performance were below the population norms, whereas their other psycho-social health was comparable to the population norms. The psycho-social scales contributed more relevant information than the physical scales to the comprehensive description of patient health after CR. All physical and psycho-social health dimensions improved except for phobic anxiety. We recommend that CR programs also include comprehensive measurement of mental health dimensions (i.e. not confined to depression and anxiety) and of social function.

## Introduction

Coronary artery disease (CAD) is responsible for 16% of the world’s deaths and the leading cause of mortality worldwide [1]. Over the past 20 years, however, advances in interventional cardiology and tertiary prevention, such as cardiovascular rehabilitation (CR), have dramatically improved the survival rates of patients with CAD [2]. Between 2000 and 2019, deaths from CAD in high-income countries fell by 16% [1].

There are contradictory reports on the outcome, in terms of improved general health and reduction in mortality, of cardiovascular rehabilitation (CR) after acute coronary syndrome (ACS). A review of 22 randomized controlled trials (RCTs) found no differences in outcomes between exercise-based CR and no exercise control for all-cause or cardiovascular (CV) mortality [3]. In contrast, a meta-analysis of 25 studies found a significantly lower hazard ratio (HR) of CV mortality (− 0.47) in the CR than in the non-CR group of ACS patients [4]. The recently updated Cochrane review of CAD (i.e., in general, with and without myocardial infarction, with and without intervention) comprised 85 trials and found, 6–12 months after CR, a slightly but not significantly reduced all-cause mortality, relative risk = 0.87; number needed to treat for an additional beneficial outcome = 75 [5]. Another meta-analysis of 24 studies pooled positive short-term effects of CR compared to “usual” care in 6 of 8 health domains of the Short Form 36 (SF-36) [6].

While several studies have been published on the somatic outcomes of CR, the literature on the effects of CR on mental and psycho-social health is sparse. Patient-reported outcomes, such as health-related quality of life (HRQL), are crucial for obtaining the patient’s perspective on the effectiveness of medical or lifestyle interventions [7]. However, little is known about how the pattern and deficits in health after an ACS and subsequent rehabilitation compared with the levels expected in the normative data from the general population.

Cardiovascular rehabilitation studies have traditionally focused on the affective dimensions of depression and anxiety. A high SCL-90R score for depression was found in 42% of CR patients [8]. Psychological distress was associated with higher rates of re-hospitalization within six months of discharge from an index hospitalization [9], and comorbid depression predicts a poorer prognosis in terms of mortality after a cardiac event [10]. Positive illness perception and life satisfaction, on the contrary, were associated with lower depression levels [11]. In the context of a life-threatening event, such as ACS, a comprehensive assessment of HRQL and mental health would therefore seem particularly relevant.

This prospective pilot cohort study aimed firstly to quantify patients’ mental and psycho-social health and HRQL at admission to and discharge from inpatient, post-ACS CR and to compare the levels with specific population norms. The norms were matched by sex, age and number of comorbid conditions. The study’s second aim was to compare the relative importance of mental and psycho-social health and of physical health in the overall assessment and to examine how the different constructs interrelate.

In addition to the traditional focus on the affective dimensions, depression and anxiety, special attention was paid in our study to phobia, interpersonal sensitivity, somatization, catastrophizing/coping, emotional role performance, and social functioning. The first hypothesis was that in these dimensions there would be substantial deficits in relation to the norm and clinically important improvements after CR. Our second hypothesis was that mental and psycho-social constructs would be at least as important as physical health dimensions for a comprehensive description of health at baseline.

## Methods

### Patients

After treatment in hospital, ACS patients were referred to the Cardiology Department of the Rehaklinik Bad Zurzach, Switzerland for a 3-to-4-week comprehensive inpatient CR program between April 2018 and October 2021.

The inclusion criterion for participation in the cardiac rehabilitation program was a confirmed diagnosis of ACS after initial acute hospital treatment, or the presence of CAD. Exclusion criteria were diagnoses other than ACS and the presence of severe comorbidities, namely severe cardiac insufficiency, valve surgery, and diseases such as terminal cancer. Also excluded from the study were patients with insufficient knowledge of the German language or psycho-intellectual abilities (e.g., dementia), which hinder participants’ understanding of the content of the rehabilitation program and assessment. The study was approved by the ethics committee of Aarau, Canton Aargau, Switzerland (EK AG 2008/026) and written informed consent was obtained from all study participants.

### Intervention

Inpatient CR is a standardized program lasting a median of three weeks and is structured according to the guidelines of the Swiss Working Group for Cardiovascular Prevention, Rehabilitation and Sports Cardiology (SCPRS). The SCPRS recommends categorizing cardiac patients in at least three different levels [12]. In order to ensure that patients’ individual needs were addressed and to form more homogenous therapy groups, four instead of three levels were established. Each patient was assigned to one of the four treatment categories according to their physical fitness, assessed at admission by a physical examination by a specially trained physiotherapist, and their performance on the six-minute walking distance (6MWD) test. If patients were completely unable to perform a 6MWD test, they were allocated to group 4. In contrast, patients who could complete the 6MWD without resting were assigned to group 3, regardless of the distance covered. On admission to the inpatient CR program no patient was assigned to the groups 1 or 2, since those patients received outpatient therapy.

Group 1 went on one-hour hiking and Nordic walking tours on hilly terrain five days a week throughout the rehabilitation program. Group 2 went on one-hour hikes on flat ground four days a week throughout the program. Group 3 patients had to be able to walk for at least 30 min on flat ground. In the therapy program, members of this group were to be trained five days a week. Additionally, the patients in groups 1, 2 and 3 had two to three ergometer training sessions per week. Group 4, to which the majority of patients were allocated at admission, were in generally poor physical shape, with reduced mobility. Initially, the focus was laid on gait exercises in individual therapies, and if the patients’ general condition permitted, training on a MOTOmed® [13], a motorized machine that enables cyclic leg movements, was carried out twice a week. Depending on the extent of their improvement over the period of CR, patients were moved up to a higher category. This depended, among other things, on their performance during the ergometer training. If they could achieve a constant performance of >40 watts over a period of 30 min, they were promoted from group 3 to group 2; with a performance of >70 watts patients moved up from group 2 to group 1.

The standard exercise program consisted of 12–18 training sessions spread over 6 days per week. In accordance with international recommendations, the focus was on aerobic endurance training [14, 15]. The training sessions comprised group endurance exercises, as well as strength and balance exercises on an individual or group basis. In groups 1–3, 39–44% of the sessions were group endurance exercises, 44% group strength and balance exercises and the remaining 12–17% were individual physiotherapy sessions. Group 4 had fewer training sessions on the whole, with 17% group endurance exercises where possible, 25% individual physiotherapy and 58% group strength and balance exercises. These basic training schemes were adapted to each patient, and passive therapies (e.g., massage or progressive muscle relaxation) were added if indicated. All patients were offered dietary counselling, psychological support and, if required, participation in a smoking cessation program. Regular ward rounds were conducted three to five times per week to exchange information within the treating team.

### Measures

Sociodemographic and disease-relevant data were gathered using a standardized questionnaire whose usefulness and validity have been established in several previous studies [16]. All necessary medical records were obtained to enable confirmation of the diagnosis, ensure fulfilment of the inclusion and exclusion criteria and evaluate the presence and number of comorbid conditions.

The Short Form 36 (SF-36) is the most-widely used questionnaire for the self-assessment of generic health and health-related quality of life [16, 17]. It is not condition-specific and allows comparison of comprehensively measured health in people with different diagnoses as well as in the healthy. We used the validated German translation of the revised version 2 [18, 19]. From a representative German general population survey (n = 6945), normative values were retrieved, which are stratified by sex, age (5-year classes), and the presence or absence of comorbid conditions [20].

The Symptom Checklist-90 Revised (SCL-90R) is one of the best-established tools for assessing psychiatric syndromes; it also normative data from the German population (n = 2025) [21, 22]. The usefulness and validity of both the SCL-90R and the SF-36 have been demonstrated specifically for rehabilitation settings [19]. The SCL-90R Depression (13 items), Anxiety (10 items), Phobic anxiety (seven items), Interpersonal sensitivity (nine items), and Somatization (12 items) scales were selected, as the other scales assess psychotic and schizophrenic symptoms only.

From the Coping Strategy Questionnaire (CSQ), the catastrophizing scale, consisting of six items, each with responses scaled by seven levels from “never” to “always”, was selected together with the two last items (control pain and decrease pain) [23, 24]. These three scales are the most responsive for the measurement of chronic pain coping [25]. The experience of acute chest pain may alter pain memory and cause fear of recurrence, and both may increase catastrophizing. Catastrophizing is an important predictive factor for pain and physical function [26].

As an examiner-based measure of functional capacity, a 6MWD was applied, one of the most frequently used, best-validated and responsive functional performance tests [27, 28]. The validated German versions of the SF-36 and the SCL90-R, together with the user’s manuals were purchased; this documentation comprises the licences for application. The CSQ is freely available and has been translated and validated by our group [24].

### Analysis

For ease of inter-scale comparison, the scoring of all the self-report instruments used was converted to the scaling system of the SF-36, i.e. 0 = worst health, maximum symptoms/disability to 100 = best health, no symptoms, full function. This means, by extension, that 0 = maximum and 100 = none on the scales of the SF-36 Bodily pain, CSQ Catastrophizing, SCL-90R Depression, SCL-90R Anxiety, etc. The walking distance of the 6MWD was quantified in meters. For the SF-36, more than 50% of the items had to be completed to determine a specific scale, which defines the instrument’s original “missing rule” [18, 19]. For the SCL-90R, it was 66.7% (two thirds) of the items per scale [16, 29]. Since no missing rules were specified in the original description of the CSQ, the requirement was similarly set at completion of more than 66.7% (two thirds) of the items, as in various earlier studies [16, 29]. All analyses were performed using the statistical software package IBM SPSS 28.0 for Windows® (SPSS Inc., Chicago, IL, USA).

Score data were described at admission (baseline) and discharge (follow-up) after CR by means and standard deviations. At both time points, the scores were compared to age-, sex- and comorbidities (present/absent)-matched population norms by standardized mean differences (SMDs). The SMD is the difference between the patient scores and the norm scores in numbers, divided by the pooled standard deviation of the patient and norm scores [30]. The SMD is equal to the test statistic of the t-test for independent samples, i.e., inter-individual differences [30, 31].

Between baseline and follow-up, intra-individual score differences were then standardized by the (baseline) effect size (ES) according to Kazis and the standardized response mean (SRM) according to Liang [32, 33]. This enables comparisons of score changes across different constructs, which are relatively independent of the baseline scores and the scaling [31]. The ES is equal to the score difference (baseline to follow-up) divided by the standard deviation of the baseline scores and can be used for effect estimates based on one-point data. The SRM is the score difference (baseline to follow-up) divided by the standard deviation of the score changes and is the intra-individual equivalent of the inter-individual SMD [31].

For all effect size parameters, ES, SRM and SMD, the 95% confidence intervals (95%-CI: parameter ± *t*-value*standard error) were given, to test whether the differences were statistically significantly different from zero (exclusion of zero by the 95%-CI) together with the corresponding *p*-value (type one error) [31]. For all types of effect size, a positive value of >0.80 is considered a large, 0.50–0.79 a moderate, 0.20–0.49 a small, and 0.00–0.19 a very small improvement. A negative ES or SRM reflects worsening between baseline and follow-up.

On the basis of our experience with the comprehensive measurement of low back pain syndrome, factor analysis with varimax rotation was performed in order to quantify and compare the importance of the mental and psycho-social health dimensions and the physical dimensions [34]. Factor analysis is a multivariate correlation analysis designed to reduce the number of dimensions, identify common constructs and explain the nature of their interrelations [35, 36]. For the extraction of the number of factors of the factor analysis, “Velicer’s minimum average partial (MAP) test” and “parallel analysis” were used [35]. By orthogonal projection of the vector of a certain scale on the common factor in the multidimensional space, this factor load reflects the correlation between the scale and the factor, the common construct [35, 36]. The explained variances of the main domains represented by the extracted factors depict the relative importance of those constructs in overall bio-psycho-social health.

Of the 17 scales examined, 14 were included for factor analysis to achieve finite data [35, 36]. Excluded were the SF-36 Mental health, because its construct is fully covered by the SCL-90R scales [35, 36] and the SF-36 General health because its construct is midway between mental and physical and is already covered by the other SF-36 and SCL-90R scales [35, 36]. The CSQ Decrease pain was also excluded, because it measures almost the same construct as the CSQ Control pain (Pearson correlation of 0.87 in the present study).

Sample sizes of n ≥ 70 make it possible to approximate the normal distribution of data generated by stochastic processes due to the central limit theorem and allow the use of parametric statistical parameters, as the SMD [37]. At n = 70, an SMD = 0.34 is statistically different from zero at the two-sided type I level *p* = 0.050 with a 95% confidence interval of (0.006, 0.670) [30]. A factor analysis should comprise at least five cases per variable included, i.e., 5*14 (scales) = 70 patients [35].

## Results

### Patients and baseline disease-relevant data (Table 1)

**Table 1:** Socio-demographic and disease-relevant data at baseline (n = 70)

Characteristics	n/mean	%/sd
Male (%)	55	79
Living alone (%)	29	41
Education (%)		
Basic schooling (8–9 years)	10	14
Vocational training	27	39
College/high school/university	33	47
Comorbidities (%)		
None	2	3
1	5	7
2	8	11
3	13	19
≥ 4	42	60
Smoking (%)	7	10
Walking/cycling (hours/week; %)		
0	5	7
< ½	14	20
½–1	31	44
> 1	20	29
Sports (hours/week; %)		
0	23	33
< 1	8	11
1–2	19	27
> 2	20	29
Working place (hours/week; %)		
0	41	59
1–21	2	3
22–41	11	16
≥ 42	16	23
Household (hours/week; %)		
0	10	14
1–21	52	74
22–41	8	11
≥ 42	0	0
Acute hospital intervention		
Aorto-coronary bypass	38	54
PTA/stenting	30	43
Medication only	2	3
BMI (kg/m^2^)	26.1	4.2
Min–max	17.1–38.6	
Age (years)	66.6	9.7
Min–max	47.5–87.6	

PTA = Percutaneous transluminal angioplasty, BMI = Body Mass Index; sd = standard deviation

Of the n = 81 patients included at baseline, 70 (86%) competed the CR program and the study’s assessment. One patient went home because of his wife’s death, 2 were re-hospitalized due to acute illness, and 8 withdrew their participation during the stay.

The typical patient was male (79%), living with a partner (71%), well educated (47% college or higher), and was, on average 66.6 years old and slightly overweight with a mean BMI of 26.1 (only 12 = 17% were obese with BMI > 30.0). Comorbid conditions were frequently present (median = 4), most did not smoke (93%) and were moderately physically active: walking/cycling for a median ½ to 1 h/week, or practicing sports for a median 1–2 h/week. The median working capacity was 0 h/week spent at the working place (most participants were in fact retired) and 7 h/week spent on household chores. More than half the patients (54%) had received aorto-coronary bypass (ACBP) surgery and 43% percutaneous transluminal (coronary) angioplasty (PTA) during acute hospital care before CR.

### Baseline health and health-related quality of life and compared to norms (Table 2)

Physical health showed moderate score levels on the SF-36 (41.0–71.8) at baseline. Physical functioning (SMD − 0.75) and Role physical (SMD − 0.90) were significantly reduced compared to the norms whereas General health were better than the norm level (SMD 0.57). The level of pain (SMD 0.32) was slightly but not significantly lower than expected by the norm. The mean 6MWD was 370.1 m.

**Table 2:** ACS patient data: baseline (before CR), follow-up (after CR) and norm data (n = 70)

			Baseline			Follow-up			ES			SRM		
									Mean	95%-CI		Mean	95%-CI	
SF-36	Score	(mean, sd)	51.7	23.3		75.9	17.2		1.04	0.83	1.24	1.20	0.96	1.43
Physical functioning	Norm	(mean, sd)	68.9	22.5		68.7	22.8							
	SMD	(mean, 95%-CI)	− 0.75	− 1.09	− 0.40	0.35	0.02	0.69						
SF-36	Score	(mean, sd)	41.0	30.4		65.7	25.6		0.81	0.59	1.04	0.85	0.62	1.09
Role physical	Norm	(mean, sd)	64.4	20.4		64.2	20.9							
	SMD		− 0.90	− 1.25	− 0.56	0.06	− 0.27	0.39						
SF-36	Score	(mean, sd)	59.6	29.3		79.0	23.1		0.66	0.47	0.86	0.80	0.57	1.04
Bodily pain	Norm	(mean, sd)	52.3	12.3		52.1	12.5							
	SMD	(mean, 95%-CI)	0.32	− 0.01	0.65	1.43	1.07	1.80						
SF-36	Score	(mean, sd)	62.1	19.8		72.8	17.1		0.54	0.33	0.75	0.61	0.38	0.85
General health	Norm	(mean, sd)	53.4	9.1		53.4	9.1							
	SMD	(mean, 95%-CI)	0.57	0.23	0.90	1.41	1.04	1.78						
SF-36	Score	(mean, sd)	54.7	20.6		70.5	18.9		0.76	0.55	0.97	0.86	0.63	1.10
Vitality	Norm	(mean, sd)	54.7	10.2		54.6	10.2							
	SMD	(mean, sd)	0.00	− 0.33	0.33	1.04	0.69	1.39						
SF-36	Score	(mean, sd)	71.8	29.5		86.3	22.6		0.49	0.28	0.71	0.55	0.31	0.78
Social functioning	Norm	(mean, sd)	81.3	7.3		81.2	7.3							
	SMD	(mean, 95%-CI)	− 0.44	− 0.77	− 0.11	0.30	− 0.03	0.63						
SF-36	Score	(mean, sd)	71.8	29.9		81.7	23.3		0.33	0.11	0.55	0.36	0.12	0.60
Role emotional	Norm	(mean, sd)	81.9	9.1		81.9	9.1							
	SMD	(mean, 95%-CI)	− 0.45	− 0.79	− 0.12	− 0.01	− 0.34	0.32						
SF-36	Score	(mean, sd)	76.1	17.5		83.2	16.7		0.40	0.19	0.61	0.45	0.21	0.69
Mental health	Norm	(mean, sd)	70.1	7.4		70.0	7.4							
	SMD	(mean, 95%-CI)	0.45	0.12	0.78	1.01	0.66	1.37						
SF-36	Score	(mean, sd)	37.3	11.0		47.3	7.5		0.91	0.71	1.12	1.05	0.81	1.28
Physical CS	Norm	(mean, sd)	40.4	7.6		40.3	7.7							
	SMD	(mean, 95%-CI)	− 0.33	− 0.66	0.01	0.91	0.57	1.26						
SF-36	Score	(mean, sd)	50.9	12.3		54.0	10.3		0.25	0.07	0.44	0.32	0.08	0.56
Mental CS	Norm	(mean, sd)	51.1	2.9		51.1	2.9							
	SMD	(mean, 95%-CI)	− 0.02	− 0.35	0.31	0.38	0.05	0.71						
SCL-90R	Score	(mean, sd)	87.7	14.9		93.0	12.7		0.35	0.23	0.47	0.68	0.44	0.92
Depression	Norm	(mean, sd)	89.8	3.5		89.8	3.5							
	SMD	(mean, 95%-CI)	− 0.20	− 0.53	0.13	0.33	0.00	0.66						
SCL-90R	Score	(mean, sd)	92.8	9.6		96.3	7.9		0.36	0.19	0.54	0.49	0.26	0.73
Anxiety	Norm	(mean, sd)	93.6	2.4		93.6	2.4							
	SMD	(mean, 95%-CI)	− 0.12	− 0.45	0.21	0.45	0.12	0.79						
SCL-90R	Score	(mean, sd)	95.0	8.2		96.0	7.7		0.12	0.00	0.25	0.23	-0.01	0.47
Phobic anxiety	Norm	(mean, sd)	96.8	1.2		96.8	1.2							
	SMD	(mean, 95%-CI)	− 0.31	− 0.64	0.02	− 0.15	− 0.48	0.18						
SCL-90R	Score	(mean, sd)	94.0	9.1		96.1	7.0		0.23	0.08	0.39	0.35	0.12	0.59
Interpersonal sensitivity	Norm	(mean, sd)	91.1	2.2		91.0	2.2							
	SMD	(mean, 95%-CI)	0.44	0.11	0.78	0.98	0.63	1.32						
SCL-90R	Score	(mean, sd)	86.2	12.3		91.8	8.2		0.45	0.29	0.61	0.67	0.44	0.91
Somatization	Norm	(mean, sd)	88.5	3.3		88.5	3.3							
	SMD	(mean, 95%-CI)	− 0.26	− 0.59	0.07	0.51	0.18	0.85						
CSQ Catastrophizing	Score	(mean, sd)	83.6	16.8		91.3	13.0		0.46	0.26	0.65	0.56	0.32	0.79
CSQ Control pain	Score	(mean, sd)	55.1	28.2		68.2	30.2		0.47	0.23	0.71	0.46	0.22	0.69
CSQ Decrease pain	Score	(mean, sd)	54.4	26.8		68.5	24.7		0.53	0.27	0.78	0.48	0.24	0.72
6MWD	meters	(mean, sd)	361.8	125.5		479.8	118.5		0.94	0.75	1.13	1.19	0.95	1.43

SF-36 = Short Form 36; CS = component summary; SCL-90R = Symptom Checklist-90 Revised; CSQ = Coping Strategies Questionnaire; 6MWT = 6 min walking test. Scaling of all scores: 0 = worst (maximal symptoms/disability); 100 = best (no symptoms/full function); exception: 6MWD (meters), sd = standard deviation for scores and norms or the lower and upper limit of the 95% confidence interval (95% CI) for the SMD, SMD = standardized mean difference between the scores and the norms; ES = effect size (according to Kazis), SRM = standardized mean difference (according to Liang), CI = confidence interval of the SMD/ES/SRM; p = type I error of the test that the SMD ≠ 0 (2-tailed, independent samples)

In the domains of mental health and psycho-social abilities, SF-36 Social functioning (SMD − 0.44) and Role emotional (SMD − 0.45) were significantly impaired whereas Vitality was not. In the SCL-90R scales Depression, Anxiety, Phobic anxiety and Somatization, the mean score levels were also slightly lower than those of the norms but the differences did not reach statistical significance (SMDs − 0.12 to − 0.31). SCL-90R Interpersonal sensitivity (SMD 0.44) was significantly higher than its population norm. On the CSQ, relatively low catastrophizing was observed (mean score 83.6; 100 = no catastrophizing), whereas the ability to control and decrease pain showed relatively low levels of coping (55.1 and 54.4, respectively; 100 = maximum control/decrease).

### Health and health-related quality of life at follow-up and compared to norms (Table 2)

The follow-up scores of most scales were higher than expected by the population norms by SMDs between 0.33 (SCL 90R depression) to 1.43 (SF-36 Bodily pain; exceptions were: SF-36 Role physical (SMD 0.06), Social Functioning (SMD 0.30), Role emotional (SMD − 0.01) and SCL-90R Phobic anxiety (SMD − 0.15).

On almost all scales, statistical significant improvements were observed between baseline and the follow-up by ES between 0.23 on SCL-90R Interpersonal sensitivity to 1.04 on SF-36 Physical functioning; exception: SCL-90R Phobic anxiety with ES 0.12. Namely, depression and anxiety improved by ES 0.35 and 0.36, coping by ES of 0.46 to 0.53, Vitality by ES 0.76, and the 6MWD by ES 0.94. The corresponding SRMs showed somewhat higher but comparable levels to those of the ESs, except on the SCL-90R Depression scale.

### Factor analysis (Table 3)

Mental health and function & pain were the dominating factors for the scores at baseline and follow-up as well as for the score differences explaining 26.3–41.1% of the variances followed by pain and function explaining 11.2–15.8%. All three models showed good fit to explain high proportions of the empiric variances (61.7–72.2%).

**Table 3:** Factor loads of baseline scores, follow-up scores and score differences (difference between baseline and follow-up scores)

	Baseline				Follow-up			Difference			
	Mental	Funct. Pain	Walking	Coping	Mental	Funct. Pain	Coping	Mental	Funct. Pain	Social	Coping
SF-36 physical functioning	0.05	0.58	**0.68**	− 0.10	0.25	**0.80**	− 0.14	0.11	**0.83**	0.04	− 0.02
SF-36 role physical	0.14	**0.72**	0.45	− 0.11	0.43	**0.72**	− 0.13	0.13	**0.76**	0.26	− 0.01
SF-36 bodily pain	0.03	**0.80**	0.06	0.08	0.26	**0.67**	0.32	− 0.25	**0.71**	0.05	0.32
SF-36 vitality	0.44	**0.66**	0.19	− 0.15	0.64	0.59	0.10	0.22	**0.74**	0.07	− 0.02
SF-36 social functioning	0.31	**0.67**	− 0.01	− 0.11	0.56	0.40	− 0.06	− 0.11	0.31	**0.77**	0.20
SF-36 role emotional	**0.73**	0.35	0.03	− 0.05	**0.65**	0.39	− 0.09	0.27	0.08	**0.80**	− 0.33
SCL-90R depression	**0.85**	0.31	0.03	− 0.02	**0.90**	0.21	0.01	**0.68**	0.16	0.46	0.09
SCL-90R anxiety	**0.74**	0.01	0.14	0.43	**0.92**	0.09	0.11	**0.66**	− 0.06	0.06	0.22
SCL-90R phobic anxiety	**0.73**	0.22	0.10	0.28	**0.85**	0.11	0.04	**0.71**	0.15	0.12	− 0.03
SCL-90R interpersonal sens	**0.88**	0.07	− 0.08	− 0.05	**0.92**	0.07	0.10	**0.78**	− 0.01	0.08	− 0.12
SCL-90R somatization	0.55	0.54	0.07	0.37	0.53	**0.60**	0.19	0.31	0.06	0.53	0.58
CSQ catastrophizing	**0.67**	0.06	0.39	− 0.22	**0.65**	0.33	0.20	0.52	0.18	− 0.26	0.16
CSQ control pain	0.03	− 0.10	− 0.01	**0.85**	0.08	0.02	**0.94**	0.25	− 0.08	0.01	**0.69**
6 Minute walking distance	0.07	0.07	**0.89**	0.09	− 0.09	0.57	0.04	− 0.11	0.14	− 0.07	0.59
Explained variance (%)	41.1	15.3	8.5	7.2	50.2	11.2	7.7	26.3	15.8	10.3	9.3
Total explained variance (%)				72.2			69.1				61.7

SF-36 = Short Form 36; SCL-90R = symptom checklist-90 revised; CSQ = coping strategies questionnaire, 6MWD = six minute walking distance, Funct. = function, sens. = sensitivity; bold are factor loads ≥ 0.60

At baseline, walking (6MWD and SF-36 Physical functioning) formed a third factor but at the follow-up, both loaded together with SF-36 Role physical and Bodily pain. Coping was also an extracted factor covering constructs different from mental health and function and pain. For the score differences between baseline and the follow-up, SF-36 Social functioning together with Role emotional was here the third extracted factor (10.3%) before coping.

### Sensitivity analysis according to the acute intervention before CR

The outcomes of the 38 patients with previous ACBP and the 30 patients having undergone PTA were compared (data not shown in detail). At baseline, SF-36 physical functioning was, in trend, better in the PTA group: mean = 58.0 (SD = 23.1) versus 47.8 (22.6), *p* = 0.071; this was also true for SF-36 Bodily pain: 68.3 (30.3) versus 55.0 (26.4), *p* = 0.057. In all the other dimensions, the differences were small and not significant (*p* > 0.200). At the follow-up, the PTA group showed a slight trend to better function on both the SF-36 Physical functioning: 80.7 (19.9) versus 73.2 (12.7), *p* = 0.063 and the SF-36 Role physical: 72.4 (27.4) versus 61.3 (23.0), *p* = 0.073. On the 6MWD the score changes between baseline and follow-up showed more effect among the ACPB subjects: plus 140.1 (71.3) meters vs PTA 93.9 (62.9) (*p* = 0.009). In addition, the ACBP group experienced a slightly greater improvement in SF-36 Vitality: 19.3 (16.9) versus 11.0 (19.4), *p* = 0.063. In summary, state and changes on all specific mental and coping scales showed no statistically significant differences between the two acute treatment groups.

## Discussion

This prospective cohort study explored the HQRL of patients admitted to the cardiac department of our rehabilitation centre after initial hospital treatment for ACS elsewhere. The study focused on the mental and psycho-social domains before and after inpatient CR and compared the levels to specific population norms.

Whereas, at baseline, physical and social function and role performance (SF-36) were significantly worse compared to the sex, age and morbidity-matched population norms, the scores on most of the scales of the mental and psycho-social domains were comparable or higher. SCL-90R Phobic anxiety was slightly but not statistically significantly worse than the population norm. The scores on the SF-36 General health, SF-36 Mental health, and SCL-90R Interpersonal sensitivity scales were significantly higher than the norms. By that, our first hypothesis that substantial normative deficits at admission to CR would be found in the mental and psycho-social dimensions was only partly confirmed. As depression and anxiety are particularly under-recognized in men, the predominantly male sample in our study might account for these findings [10].

Nonetheless, even in the domains where the baseline scores were higher than the norms and also exceeded our expectations, there were significant improvements at discharge. Factor analysis revealed that mental and psycho-social health is a key domain in the comprehensive assessment of health before and after CR. It is more important than the domain of pain and function. Sensitivity analysis showed that the state and changes of health and HRQL were comparable between groups having received different types of pre-CR acute intervention (ACBP and PTA), and especially so in the mental health and coping dimensions. The only difference found was on the 6MWD, where the post ACBP improved more than post PTA group.

A comparable study found a considerable number of symptoms on the SCL-90 scales of Depression, Anxiety, and Somatization among patients entering CR [8]. However, it is not clear to which norms the retrospective, cross-sectional data of the study were compared – certainly not to individual norms according to sex and age—and the SCL-90 original scores were not reported. The same is true for the SCL-90 scores on admission to CR of another cross-sectional cohort, where, in contrast to the study of Kolmann et al., the patients reported less depression and somatization than expected from the global US norm [8, 9]. One possible explanation for the high baseline health levels of our patients may be that most reported relatively high activity levels, whether in walking, sport or work.

After CR at our clinic, most patients’ health dimensions showed statistically significant improvements beyond the population norm levels (SMDs up to 1.43 for SF-36 Bodily pain) with standardized ESs up to 1.04 (SF-36 Physical functioning) on physical health, 0.94 on the 6MWD, and up to 0.76 (SF-36 Vitality) on the mental health scales. All scores on the SCL-90R (except Phobic anxiety), and on all three CSQ coping scales also showed improvement between baseline and follow-up. Broadening the measurement of mental health dimensions beyond the classical focus on depression and anxiety provided additional important information on CR after ACS.

Two recent meta-analyses of SF-36 data, in which there was a partial overlap of the individual studies summarized, showed comparable score levels to our post-CR follow-up scores [6, 38]. There was wide variation in the effect differences between the single studies, so that the pooled effects differ considerably between the two meta-analyses. Both reviews pooled the follow-up score differences between exercise and control group instead of intra-individual score differences between the two groups, an approach that inhibits direct comparison of the effect levels to score changes. However, the largest differences were detected on SF-36 Role physical, followed by Physical functioning, Social functioning and Vitality, whereas on Role emotional and Mental health, the differences were relatively smaller. The SF-36 score differences observed in our patients are consistent with the pattern described above. A similar pattern can be seen in a third meta-analysis, which, in addition to pooling studies that overlapped with the two above-mentioned meta-analyses, quantified the score changes by means of SMDs [39]. SF-36 Physical functioning showed significance in favour of the exercise group, whereas the Physical component summary (PCS), which includes all other scales, did not. Due to the considerable variance of the effects among the studies included, in all three domains (physical, mental, social), non-exercise or psychologically treated groups showed significant effects but not the exercise groups.

Our second hypothesis that the mental and psycho-social domain is more important than physical symptoms/abilities was consistently true for all three factor analyses (baseline, follow-up, score differences), as shown by explained variances up to 41.1% compared to function and pain, which explained up to 26.3%. Interestingly, the walking test formed a third factor at baseline [26]. Self-efficacy/management, which is directly and indirectly addressed in CR, is known to be an important positive prognostic factor for mental health and HQRL after ACS [40].

The main strength of our study consists in the fact that for two of the best tested and validated instruments for measuring HQRL, namely the SF-36 and SCL-90R, patient scores were for the first time compared to age- and sex- matched population norms. For the SF-36 the comparison extended to the presence or absence of comorbid conditions. Standardized scaling and quantification of differences to the norm and of changes between baseline and follow-up allowed comparison across various constructs. Explorative factor analysis quantified the contribution of the main construct domains to the comprehensive measurement of bio-psycho-social health.

A limitation of our study was the small sample size of n = 70. However, larger sample sizes tend to overpower the effect estimates by narrowing the 95% confidence intervals. As reported in a large body of literature, changes below 0.30 (ES or SRM) are, on the group level, subjectively imperceptible, being smaller than the minimal clinically important differences [31]. The baseline data suggest that our patients may have been in better health than the participants in comparable studies. However, high baseline scores tend to show lower effects than lower baseline scores as a result of the (opposite) regression-to-the mean effect [31]. Thus, the improvements are more likely to be under- than overestimated.

## Conclusions

This prospective cohort study showed that, compared to individually specific population norms, patients admitted to post-ACS CR evidenced baseline deficits in physical but not in psycho-social health domains. At the follow-up, after CR, significant improvements were found in all scores except in phobia. A broader, more comprehensive measurement of bio-psycho-social health should comprise not only depression and anxiety but in particular somatization and social participation. For clinical purposes, at least two mental health scales and at least one coping scale can be recommended for a comprehensive and specific assessment of health and HLRQ.

## Data Availability

All data and material are freely available. Please contact the corresponding author with data requests.

## Abbreviations

6MWD: Six-minute walking distance test
ACBP: Aorto-coronary bypass graft surgery
ACS: Acute coronary syndrome
BMI: Body Mass Index
CAD: Coronary artery disease
CI: Confidence interval
CR: Cardiac rehabiliation
CV: Cardio vascular
CSQ: Coping strategy questionnaire
EK AG: Ethics (K)committee of canton Aargau
ES: Standardized effect size (according to Kazis 1989)
HR: Hazard ratio
HRQL: Health-related quality of life
MAP: Velicer’s minimum average partial test
MCID: Minimal clinically important difference
MCS: Mental component summary (of the SF-36)
N: Number (of patients)
PTA: Percutanenous transluminal (coronary) angioplasty/stenting
PCS: Physical component summary (of the SF-36)
RCT: Randomized controlled trial
SCL-90R: Symptom checklist-90 revised
SCPRS: Swiss working group for cardiovascular prevention, rehabilitation and sports cardiology
SF-36: Short Form 36
SMD: Standardized mean difference
SRM: Standardized response mean (according to Liang 1990)
